# Association between Brain Injury Markers and Testosterone in Critically-Ill COVID-19 Male Patients

**DOI:** 10.3390/microorganisms10112095

**Published:** 2022-10-22

**Authors:** Daria Tokic, Marijana Mikacic, Marko Kumric, Tina Ticinovic Kurir, Iva Rancic-Vidic, Dinko Martinovic, Josipa Bukic, Josip Vrdoljak, Ivan Kresimir Lizatovic, Sanda Stojanovic Stipic, Daniela Supe Domic, Josko Bozic

**Affiliations:** 1 Department of Anesthesiology and Intensive Care, University Hospital of Split, 21000 Split, Croatia; 2 Intensive Care Unit, Department of Internal Medicine, University Hospital of Split, 21000 Split, Croatia; 3 Department of Pathophysiology, School of Medicine, University of Split, 21000 Split, Croatia; 4 Department of Endocrinology, Diabetes and Metabolic Diseases, University Hospital of Split, 21000 Split, Croatia; 5 Department of Pharmacy, School of Medicine, University of Split, 21000 Split, Croatia; 6 Department of Acute Respiratory Infections, University Hospital for Infectious Diseases Dr. Fran Mihaljevic, 10000 Zagreb, Croatia; 7 Department of Health Studies, University of Split, 21000 Split, Croatia; 8 Department of Medical, Laboratory Diagnostics, University Hospital of Split, 21000 Split, Croatia

**Keywords:** GFAP, UCH-L1, SARS-CoV-2, COVID-19, testosterone, neurovascular damage

## Abstract

Accumulating data suggest that various neurologic manifestations are reported in critically-ill COVID-19 patients. Although low testosterone levels were associated with poor outcomes, the relationship between testosterone levels and indices of brain injury are still poorly understood. Therefore, we aimed to explore whether testosterone levels are associated with glial fibrillary acidic protein (GFAP) and ubiquitin carboxy-terminal hydrolase L1 (UCH-L1), biomarkers of brain injury, in patients with a severe form of COVID-19. The present study was conducted on 65 male patients aged 18–65 with severe COVID-19. Blood samples were collected at three time points: upon admission to ICU, 7 days after, and 14 days after. In patients with neurological sequels (*n* = 20), UCH-L1 serum concentrations at admission were markedly higher than in patients without them (240.0 (155.4–366.4) vs. 146.4 (92.5–243.9) pg/mL, *p* = 0.022). GFAP concentrations on admission did not differ between the groups (32.2 (24.2–40.1) vs. 29.8 (21.8–39.4) pg/mL, *p* = 0.372). Unlike GFAP, UCH-L1 serum concentrations exhibited a negative correlation with serum testosterone in all three time points (r = −0.452, *p* < 0.001; r = −0.430, *p* < 0.001 and r = −0.476, *p* = 0.001, respectively). The present study suggests that the traumatic brain injury biomarker UCH-L1 may be associated with neurological impairments seen in severe COVID-19. Moreover, a negative correlation between UCH-L1 and serum testosterone concentrations implies that testosterone may have a role in the development of neurological sequels in critically-ill COVID-19 patients.

## 1. Introduction

Since the initial coronavirus disease 2019 (COVID-19) outbreak in China in December 2019, COVID-19 has risen to become the biggest medical issue of modern medicine henceforth [1,2]. Aside from the well-established respiratory failure observed in critically-ill COVID-19 patients, clinical experience and accumulating data suggest that various neurologic manifestations are also seen in this population [3,4]. Headache, dizziness, myalgia, and almost pathognomonic anosmia are among the general neurologic symptoms documented in COVID-19, whereas acute ischemic stroke, encephalopathy, multifocal necrotizing cerebral hemorrhages, and delirium have all been reported in hospitalized patients [5,6]. The pathophysiologic background of neurological manifestations in COVID-19 is dual. Firstly, owing to SARS-CoV-2 neurotropism, neurological disorders may arise as a consequence of direct viral effects. In addition, disturbance of the blood-brain barrier and vasculopathy may also potentiate shedding of SARS-CoV-2 in the brain [7]. Conversely, it has been suggested that neurologic manifestations in critically ill COVID-19 patients may also be a result of increased host-immune response and insufficient cerebral oxygen delivery due to cardiorespiratory failure [8,9]. Furthermore, several molecules involved in the coagulation cascade, such as von Willebrand Factor and lupus anticoagulant, have been found to play a crucial role in prothrombotic complications in COVID-19, leading to cerebral microvasculature damaging and late neuropathic and neurologic sequelae [10]. It was found that these molecules promote platelet adhesion and aggregation, induce the release of tissue factor, and activate the coagulation cascade, leading to a sometimes-irreversible hypercoagulable state.

Despite the fact that a vast amount of resources was utilized for research of COVID-19, there are still no objective clinical measurements for the quantification of potential neurological deficit in this setting. Therefore, several molecules emerged as potential biomarkers of neurological disorders seen in COVID-19. Glial fibrillary acidic protein (GFAP) is an intermediate filament mainly present in mature astrocytes [11]. Accumulated evidence suggests that GFAP proteins’ abnormal expression occurs in traumatic brain injury, neuroinflammation, neurodegeneration, and psychiatric disorders [12,13]. Furthermore, a body of data implies that Ubiquitin carboxy-terminal hydrolase L1 (UCH-L1), an enzyme abundantly expressed in the cytoplasm of neurons, is associated with the severity of brain injury [14,15]. Moreover, both of these proteins were recently associated with delirium in patients with a severe form of COVID-19 [16]. Additionally, several other molecules have been investigated as potentially involved in brain injury, such as Neuropilin-1 [17]. This widespread signal protein involved in several systems, among them olfactory epithelium, has found to be a certain “gate” for SARS-CoV-2 virus spreading. Furthermore, this pathway is not only allowing the virus to cause anosmia, a pathognomonic symptom of the disease, but to facilitate infiltration to the CNS, potentially causing further damage to the brain.

The development of hypogonadism in male patients with COVID-19 has been extensively explored. According to available evidence, an inflammatory response in COVID-19 suppresses the reproductive hormone axis, and a lack of testosterone further stimulates the release of pro-inflammatory cytokines, thus closing a vicious cycle [18]. Consequently, hypogonadism foreshadows a fatal course of the SARS-CoV-2 infection [19]. Although low testosterone levels were associated with poor outcomes, relationships between testosterone levels and indices of brain injury are still poorly understood.

In the present study, therefore, we aimed to explore the dynamic of GFAP and UCH-L1 during the ICU stay of patients with severe COVID-19. Furthermore, we aimed to explore whether testosterone levels are associated with GFAP and UCH-L1, showing biomarkers of neurological disorders in male patients with a severe form of COVID-19. Finally, we explored whether GFAP and UCH-L1 levels are higher in patients who developed neurological sequels during in-hospital stay.

## 2. Materials and Methods

### 2.1. Study Design and Setting

This single-center prospective observational study was performed at the University Hospital of Split intensive care unit (ICU) from January 2022 to May 2022. The study has the approval of the Ethics Committee of the University Hospital of Split (Class 500-03/21-01/185, Number: 2181-147/01/06-M.S.-21-02, Split, Croatia, 22 December 2021) and was conducted according to the ethical principles of the 2013 Declaration of Helsinki. All participants gave their informed agreement for participating before they were enrolled in the study.

### 2.2. Study Participants

The study enrolled 65 male patients aged 18–65 who were treated from a severe form of COVID-19 at the ICU of the University Hospital of Split. All of the included patients were intubated and mechanically ventilated on the admission to the ICU. The standard references for endotracheal intubation consisted of: the protection of the airway, severe decompensated respiratory acidosis (pH < 7.2), and critical hypoxemia (PaO_2_ < 50 mmHg or SpO_2_ < 90%), regardless of maximal noninvasive respiratory methods. The exclusion criteria were: female sex, active malignant illness in the past year, the presence of autoimmune disease, neuromuscular diseases, cerebrovascular accidents, previously diagnosed hypogonadism, heart failure, liver failure, and renal failure.

### 2.3. Clinical Evaluation

Sociodemographic traits and data relevant for the study (past medical history including current and chronic therapy, comorbidities, period of time from the first symptoms of the disease to the hospital admission, following time from hospital admission to ICU admission) were assembled from the hospital records for every included patient. From the admission to the ICU, vital signs and SpO_2_ were constantly monitored. BMI was calculated by formula (the body weight (kg) being divided by height-squared (m^2^)) using the most recent medical records.

Diagnosis of COVID-19 was based on the clinical presentation, a positive nasopharyngeal real-time reverse transcription polymerase chain reaction (RT-PCR) SARS-CoV-2 test, and radiological evidence of pneumonia. During the time of the patient’s ICU admission, the SARS-CoV-2 B.1.617.2 (Delta) variant was dominant in Croatia [20]. Neurological sequels were either self-reported by patients or recognized by attending a physician after extubation, and subsequently critically assessed by the attending neurologist.

### 2.4. Laboratory Evaluation

Upon admission to the ICU, the standard laboratory panel (including white blood cells, complete blood count, C-reactive protein, urea, creatinine, liver transaminases, blood glucose, cardiac-specific troponin, and D-dimers) and arterial blood gas variables were measured, and henceforward daily. Blood samples for measurement of testosterone levels were obtained upon admission to ICU and then subsequently at days 7 and 14 after ICU admission. All of the aforementioned laboratory parameters were measured according to the standard laboratory procedures, by an experienced biochemist.

Peripheral venous blood samples for GFAP and UCH-L1 measurement were collected at 3 points in time: at the admission to the ICU, 7 days, and 14 days after the ICU admission, unless the patient was transferred to another hospital department, discharged, or deceased. The samples of blood were centrifuged at 3000× *g* for 10 min, aliquoted and subsequently stored at −80 °C. When they needed to be evaluated, samples were melted at room temperature. The concentrations of GFAP (Reagent Kit: 04W17; Abbott, Chicago, IL, USA) and UCH-L1 (Reagent Kit 04W19; Abbott, Chicago, IL, USA) in the serum were determined using chemiluminescent microparticle immunoassays (CMIA). The minimum limit of detection was 2.2 ng/L for GFAP and 16.1 ng/L for UCH-L1. The level of total testosterone was determined using immunochemistry assay (Roche Diagnostics GmbH, Mannheim, Germany). Our qualified biochemist conducted all the laboratory tests according to the manufacturer’s instructions.

### 2.5. Statistical Analysis

Data analyses were performed using the statistical software SPSS Statistics for Windows^®^ (version 28.0, IBM, Armonk, NY, USA) and SigmaPlot for Windows^®^ (version 14.0, Systat Software Inc., San Jose, CA, USA). Qualitative data were shown as whole numbers (N) and percentages (%), while quantitative data were shown as mean ± standard deviation (SD) or median (interquartile range). The normality of distribution was estimated using the Kolmogorov–Smirnov test. The comparison of categorical variables was conducted using the chi-squared (χ^2^) test or Fisher’s exact test, as appropriate. Depending on the data distribution, the comparison of quantitative data was conducted with either the Student’s t-test or the Mann–Whitney U test. The dynamics of testosterone, UCH-L1, and GFAP serum levels throughout ICU stay were assessed using the Friedman test. Finally, the correlation between serum testosterone levels and UCH-L1 was explored using Spearman rank correlation analysis. The significance was set at *p* < 0.05 for all comparisons.

## 3. Results

Patients who developed neurological sequels during in-hospital stay following a severe form of COVID-19, had a significantly longer duration of mechanical ventilation and ICU stay in comparison to counterparts who did not develop such symptoms (*p* = 0.014 and *p* = 0.010, respectively). Patients who developed neurological sequels also had significantly higher hs-TnI concentrations (*p* = 0.021) and testosterone at day 14 after ICU admission (*p* = 0.046). Other baseline characteristics of interest are presented in Table 1.

**Figure 1:**
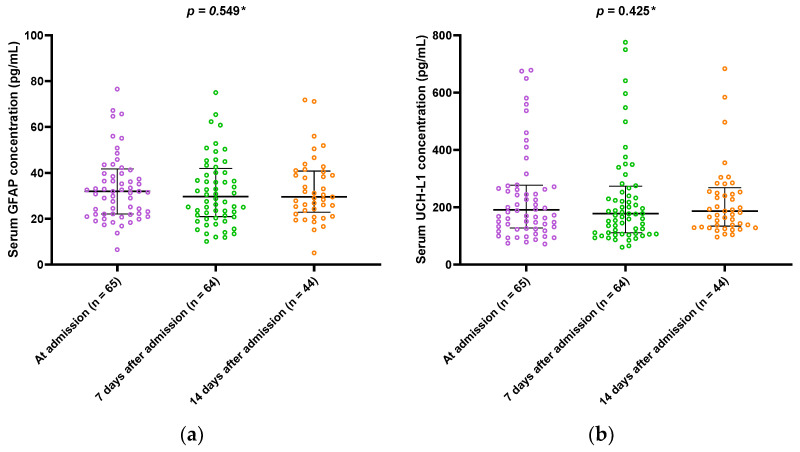

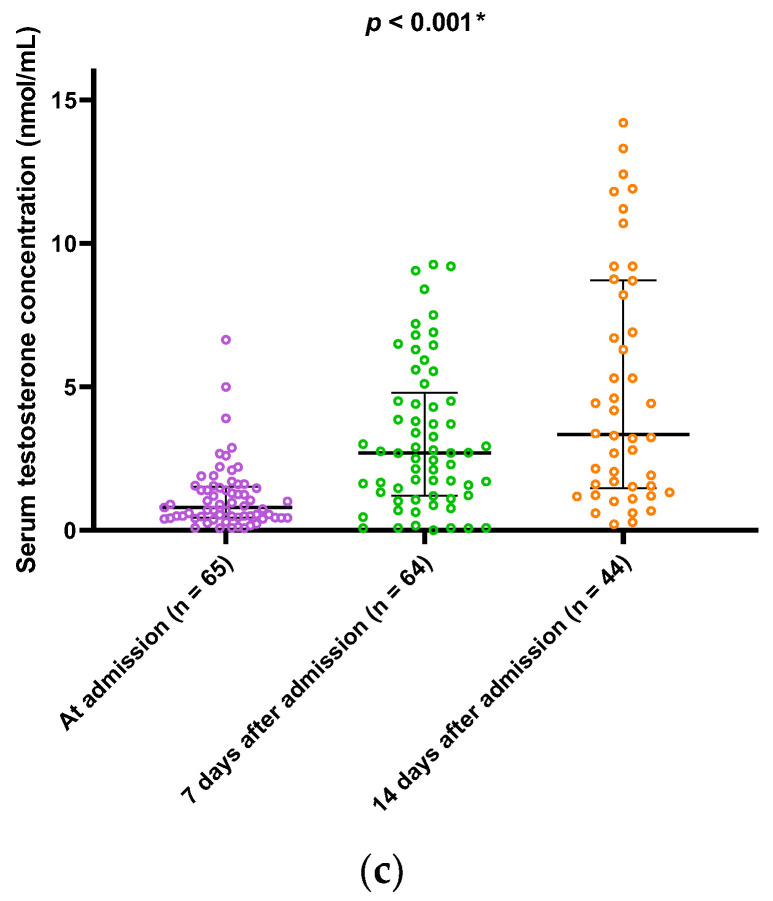
Dynamics of brain injury biomarkers and testosterone during the ICU stay in patients with severe COVID-19: (**a**) dynamics of GFAP during ICU stay; (**b**) dynamics of UCH-L1 during ICU stay. (**c**) Dynamics of serum testosterone during ICU stay; abbreviations: GFAP: glial fibrillar acidic protein; UCH-L1: Ubiquitin carboxy-terminal hydrolase L1; ICU: intensive care unit. Data are presented in the median and interquartile range. * Friedman test.

In the present study, no dynamic of GFAP or UCH-L1 serum concentrations was observed throughout the ICU stay of patients with severe form of COVID-19, *p* = 0.549 and *p =* 0.425, respectively (Figure 1a,b). On the other hand, testosterone levels increased steadily during their ICU stay (*p <* 0.001) (Figure 1c).

**Figure 2:**
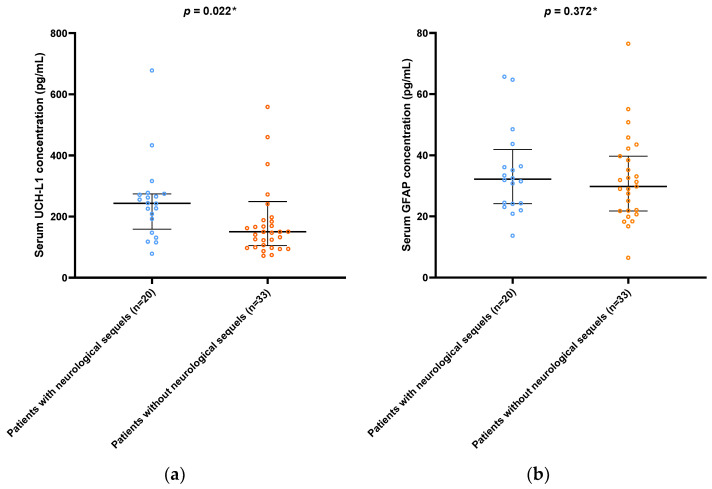
Comparison of UCH-L1 and GFAP serum concentrations on ICU admission between patients with and without neurological sequels: (**a**) UCH-L1; (**b**) GFAP. Abbreviations: UCH-L1: Ubiquitin carboxy-terminal hydrolase L1; GFAP: glial fibrillar acidic protein; ICU: intensive care unit. Data are presented in the median and interquartile range. * Mann–Whitney U test.

In patients with neurological sequels (*n* = 20), UCH-L1 serum concentrations on admission were markedly higher than in patients without them (146.4 (92.5–243.9) pg/mL vs. 240.0 (155.4–366.4) pg/mL, *p* = 0.022) (Figure 2a). On the other hand, GFAP concentrations on admission did not differ between the two groups of interest (32.2 (24.2–40.1) pg/mL vs. 29.8 (21.8–39.4) pg/mL, *p* = 0.372) (Figure 2b).

**Figure 3:**
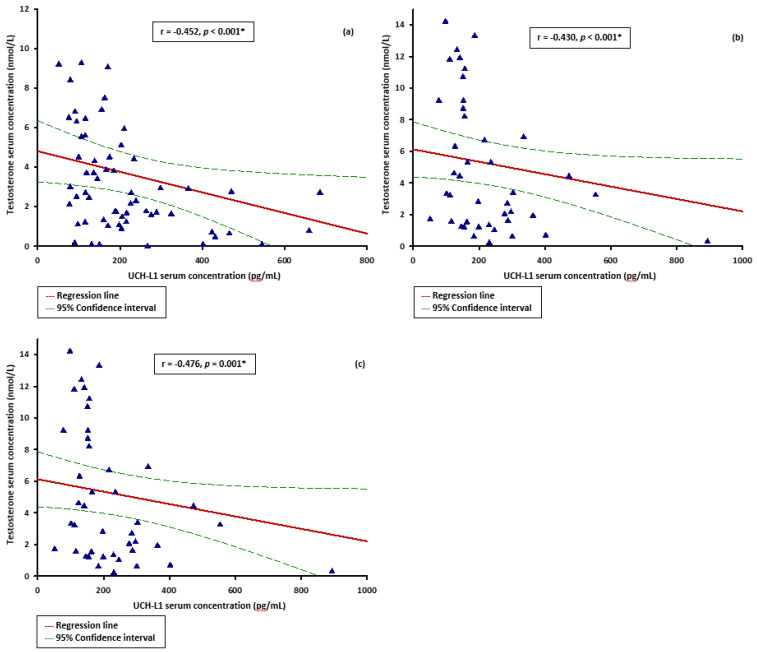
Correlation between UCH-L1 and serum testosterone concentrations at different time points of the ICU stay: (**a**) correlation between UCH-L1 and serum testosterone on admission to ICU; (**b**) correlation between UCH-L1 and serum testosterone 7 days after admission; (**c**) correlation between UCH-L1 and serum testosterone 14 days after admission. Abbreviations: UCH-L1: Ubiquitin carboxy-terminal hydrolase L1; ICU: intensive care unit. * Spearman rank correlation.

UCH-L1 serum concentrations exhibited a negative correlation with serum testosterone in all three time points (r = −0.452, *p* < 0.001; r = −0.430, *p* < 0.001 and r = −0.476, *p* = 0.001, respectively) (Figure 3a–c). Conversely, GFAP serum concentrations did not correlate with serum testosterone in any of the three time points (r = 0.091, *p* = 0.486; r = 0.111, *p* = 0.399; r = −0.190, *p* = 0.229). In addition, unlike UCH-L1 (r = 0.147, *p* = 0.245), GFAP serum levels are positively correlated with age (r = 0.397, *p* = 0.002).

## 4. Discussion

In the present study, we demonstrated that there was no significant dynamic of brain injury biomarkers UCH-L1 and GFAP in a 2-week period of ICU stay in male patients with a severe form of COVID-19. Furthermore, for the first time, we established a negative correlation between UCH-L1 and serum testosterone levels in the above-noted population. However, no such correlation was found between GFAP and testosterone. Finally, our study demonstrated that among patients who survived a severe form of COVID-19, those with neurological sequels have significantly higher UCH-L1 serum concentrations.

These results may be explained in light of the existing evidence. In a previous study, Cooper et al. explored the difference in GFAP and UCH-L1 serum levels between 27 patients with severe COVID-19 and 19 ICU controls [16]. The authors demonstrated that GFAP but not UCH-L1 levels were higher in the COVID-19 subgroup even after an adjustment for age and sex. Furthermore, in concordance with our results, they also established a positive correlation between GFAP serum concentration and age, and found no significant fluctuations of these brain injury markers during the two-week ICU stay. The most important result reported by the authors was a positive correlation between serum GFAP and UCH-L1 with Intensive Care Delirium Screening Checklist (ICDSC), a practical validated metric which is used to quantify ICU-related delirium. Notably, moderate positive correlation has been established in both the severe COVID-19 group and ICU controls. Conversely, in a separate study on COVID-19 patients, GFAP levels were elevated in comparison to controls, but declined after a mean period of 11.4 days [21]. In addition, the same study also showed that GFAP is higher in a severe form rather than in a moderate form of COVID-19, and demonstrated a positive correlation between GFAP and age. The discrepancy with the aforementioned study in GFAP serum dynamic could be explained by different population characteristics and difference in time points in which measures were obtained. Finally, Kanberg et al. explored GFAP serum levels during long-term follow-up (median of 225 days) and demonstrated that after an initial increase, GFAP serum levels return to normal [22]. Additionally, the authors found no association between higher concentrations of GFAP during the acute phase, and post-infectious neurological symptoms at a follow-up visit.

It has been well-established so far that neurologic dysfunction is commonly present in severe COVID-19 [23,24,25,26]. In the GCS-Neuro COVID study, a large multicenter international study that enrolled over 3500 patients, as many as 80% reported a neurologic manifestation, with the most common being encephalopathy [27]. The pathophysiological background explaining this injury occurs via multiple distinct pathways with poorly established relative contributions. Specifically, SARS-CoV-2 may invade the CNS by various mechanisms, including trans-synaptic transfer, olfactory nerve entry, vascular endothelial infection, and leukocyte transmigration across the blood-brain barrier [28]. Subsequently, the interaction of the virus and ACE2 in neuronal tissues initiates a cycle of viral budding accompanied by neuronal damage even in the absence of substantial inflammation [29]. In addition, as SARS-CoV-2 enters neurons, it downregulates ACE2 and disrupts a balance between ACE1 and ACE2, resulting in excessive vasoconstriction and disrupted cerebral autoregulation, thus contributing to cerebrovascular complications [29]. On an important note, the investigation of COVID-19-related neurologic manifestations is somewhat limited because these manifestations are often indistinguishable from exacerbations of pre-existing neurological and psychiatric conditions, especially in the elderly population [30]. Apart from traumatic brain injury, accumulating data suggest that both GFAP and UCH-L1 are increased in non-traumatic pathologies as well. Specifically, these biomarkers have been explored in the setting of various neurodegenerative diseases, such as Alzheimer’s and Parkinson’s, but also neuroinflammation [31,32]. As it has been established that SARS-CoV-2 may elicit the pro-inflammatory microglia phenotype, it is presumed that the elevation of brain injury biomarkers in COVID-19 may foreshadow neuroinflammation [33].

Notably, in the present study, serum testosterone levels were lower than the lower reference limit of our population (6.68 nmol/L) in all three measurement points. Several recent studies have also showed that there is a significant testosterone reduction in patients with a severe COVID-19 disease [34,35,36]. This lower testosterone expression could be due to the cortisol-induced suppression through the activation of the hypothalamic-pituitary-adrenal (HPA) which leads to higher cortisol levels in COVID-19 patients [37]. However, on the other hand, it was also established that testosterone suppresses cortisol production and it is possible that the low testosterone is actually the cause of the stress-induced HPA activation [38]. Nevertheless, it is important to highlight that this low testosterone outcome is also possibly a consequence of the hypothalamic–pituitary–gonadal axis (HPG) impairment due to the high dosage of steroids as a therapy for the severe COVID-19. These high doses of steroids are a known cause of the gonadotropin hormone-releasing hormone (GnRH) dysregulation [39]. Additionally, previous studies have showed that systemic inflammatory diseases could possibly lead to a HPG axis impairment [40,41]. Furthermore, patients with neurological sequels had lower testosterone after a 2-week stay in the ICU, but not on admission, or 7 days after, even though a tendency towards lower values has been observed. It has been suggested that testosterone is a two-way street in the setting of COVID-19 [42]. Namely, although increased testosterone concentrations could increase susceptibility to SARS-CoV-2, and portend worse outcomes by up-regulating the expression of transmembrane protease serine 2 (TMPRSS2), testosterone is also known to attenuate oxidative stress and endothelial dysfunction, thus dampening the aberrant immune response operating in severe COVID-19. So far, to the best of our knowledge, no studies have reported an association between serum testosterone levels and biomarkers of brain injury. Nevertheless, as available evidence suggests that baseline levels of testosterone may increase viral entry while paradoxically providing a relative protection from the hyperreactive immune state that drives mortality, we may hypothesize that patients with lower testosterone levels are more susceptible to brain injury observed in severe COVID-19. Such association, however, should be explored in consideration with the rest of the hormones that constitute HPG axis and on a larger sample.

The present study has several limitations. Firstly, the study was a single-center trial, and included a fairly limited number of Caucasian male patients. The rationale for including an only-male population was to explore the relationship between testosterone and neurological manifestations in severe COVID-19. Furthermore, we were not able to eliminate all of the confounding effects which could have interfered with our results. Most notably, the mortality of several included patients could have possibly affected our outcomes. Moreover, a control group of non-COVID-19 ICU patients was not included in the present analysis. Lastly, we have conducted laboratory analyses regarding only the total testosterone, while the free testosterone could have given us some more insights.

## 5. Conclusions

In summary, the present study suggests that the traumatic brain injury biomarker UCH-L1 may be associated with neurological impairments seen in severe forms of COVID-19. On the other hand, a negative correlation between UCH-L1 and serum testosterone concentrations suggests that testosterone may be a protective factor for the development of neurological sequels in critically-ill COVID-19 patients. Regardless, there is a possibility that the elevation of testosterone is actually an epiphenomenon and should only be considered as a marker of brain damage. If these findings prove to be correct in larger multicentric studies, brain injury biomarkers such as UCH-L1 could be used as a prognostic factor reflecting the occurrence of neurological deficits in severe forms of COVID-19.

## Figures and Tables

**Table 1:** Characteristics of patients at admission to the intensive care unit.

Variables	Total (*n* = 65)	In-Hospital Neurological Sequels		*p*
		No (*n* = 45)	Yes (*n* = 20)	
**Age (years)**	54.1 ± 7.6	53.4 ± 8.3	55.0 ± 7.8	0.477 *
**BMI (kg/m^2^)**	28.7 ± 3.3	28.8 ± 4.5	28.6 ± 3.3	0.851 *
**Disease duration at admission to ICU (days)**	9.2 ± 2.2	8.5 ± 3.1	9.3 ± 2.2	0.293 *
**Duration of hospitalization (days)**	16 (14–23)	16 (14–20)	20.5 (14–32)	0.199 ^†^
**ICU length of stay (days)**	13.2 ± 7.9	9.4 ± 2.9	14.3 ± 6.2	0.010 *
**Duration of mechanical ventilation (days)**	9.3 ± 7.6	7.1 ± 4.5	12.4 ± 6.6	0.014 *
**Fully vaccinated**	33 (50.8%)	22 (48.9%)	11 (55.0%)	0.825
**Deceased during ICU stay**	12 (18.5%)	12 (100%)	0 (0%)	<0.001 ^‡^
**In-hospital neurological sequels**	20 (30.8%)	N/A	20 (100%)	N/A
**Paraesthesia**	5 (7.7%)	N/A	5 (25%)	N/A
**Paresis**	8 (12.3%)	N/A	8 (40%)	N/A
**Incontinence**	3 (4.6%)	N/A	3 (15%)	N/A
**Anosmia and/or ageusia**	4 (6.2%)	N/A	4 (20%)	N/A
**Comorbidities**				
**Smoking (*n*, %)**	4 (6.2%)	3 (6.7%)	1 (5%)	1.000 ^§^
**Arterial hypertension (*n*, %)**	18 (27.7%)	14 (31.1%)	4 (20%)	0.549 ^§^
**Diabetes mellitus (*n*, %)**	4 (6.2%)	2 (4.4%)	2 (10%)	0.581 ^§^
**Dyslipidemia (*n*, %)**	42 (64.6%)	27 (60%)	15 (75%)	0.243 ^‡^
**Laboratory parameters**				
**Hemoglobin (g/L)**	133.4 ± 11.7	134.8 ± 12.1	132.8 ± 12.3	0.645 *
**Platelets (×10^9^/L)**	243.7 ± 85.6	263.2 ± 82.6	221.3 ± 89.1	0.123 *
**WBC (×10^9^/L)**	9.7 ± 3.5	10.1 ± 3.3	8.8 ± 4.4	0.222 *
**SaO_2_ (%)**	92.9 (91.3–96.0)	90.9 (90.3–96.0)	94.0 (87.6–95.5)	0.501 ^†^
**pH (units)**	7.36 ± 0.07	7.36 ± 0.08	7.34 ± 0.06	0.734 *
**pO_2_ (kPa)**	5.8 ± 1.0	5.7 ± 1.0	5.9 ± 0.7	0.508 *
**pCO_2_ (kPa)**	9.9 ± 2.1	10.0 ± 3.3	9.8 ± 2.0	0.858 *
**HCO_3_^−^ (mmol/L)**	25.7 ± 2.2	26.0 ± 2.5	25.4 ± 2.0	0.652 *
**CRP (mmol/L)**	97.5 ± 62.8	97.1 ± 65.6	94.9 ± 39.2	0.459 *
**D-dimers (mg/L)**	2.5 (1.6–4.2)	2.3 (1.3–4.2)	3.1 (1.9–6.7)	0.422 ^†^
**hs-TnI (ng/L)**	12.9 (6.4–28.3)	9.8 (4.8–14.7)	21.2 (12.8–31.7)	0.021 ^†^
**Blood glucose (mmol/L)**	9.7 ± 3.2	9.5 ± 2.4	10.6 ± 5.1	0.324 *
**Urea (mmol/L)**	8.2 ± 2.6	7.8 ± 2.2	8.9 ± 4.5	0.255 *
**Creatinine (mmol/L)**	80.3 ± 17.6	76.5 ± 14.4	83.2 ± 27.4	0.111 *
**Testosterone (nmol/L)**				
**On admission**	0.81 (0.43–1.55)	1.24 (0.50–1.49)	0.55 (0.43–1.57)	0.254 ^†^
**After 7 days**	2.70 (1.22–4.65)	3.35 (2.50–7.20)	2.80 (1.66–4.50)	0.146 ^†^
**After 14 days**	3.34 (1.52–8.70)	10.70 (3.28–12.03)	4.52 (2.15–6.90)	0.046 ^†^

Tables may have a footer. Data are expressed as mean ± SD, number (%) or median (interquartile range). * Student’s *t*-test, ^†^ Mann–Whitney U test, ^‡^ Chi-square test, ^§^ Fisher’s exact test. Abbreviations: CRP: C-reactive protein; HCO3−: bicarbonates; pO_2_: partial pressure of oxygen; pCO_2_: partial pressure of carbon dioxide; SaO_2_: oxygen saturation in arterial blood; WBC: white blood cells; hs-TnI: high-sensitivity troponin I.

## Data Availability

The data presented in this study are available on request from the corresponding author. The data are not publicly available because some of the data set will be used for further research.
